# Neurofibromin expression by normal salivary glands

**DOI:** 10.1186/s13005-021-00256-4

**Published:** 2021-02-18

**Authors:** Eloá Borges Luna, Pâmella Pinho Montovani, Rafaela Elvira Rozza-de-Menezes, Karin Soares Cunha

**Affiliations:** 1 Neurofibromatosis National Center (Centro Nacional De Neurofibromatose, CNNF), Rio De Janeiro, RJ Brazil; 2 Postgraduate Program in Pathology, School of Medicine, Universidade Federal Fluminense, Niterói, RJ Brazil; 3 Department of Pathology, School of Medicine, Universidade Federal Fluminense, Niterói, RJ Brazil; 4 Graduate Program in Pathology, School of Medicine, Hospital Universitário Antônio Pedro, Universidade Federal Fluminense, Avenida Marquês do Paraná, 303, 4º andar, sala 01 – Centro, 24033900 Niterói, RJ Brazil

**Keywords:** Neurofibromatosis 1, Neurofibromin, Salivary glands, Hyposalivation

## Abstract

**Introduction:**

Neurofibromin, a protein encoded by the *NF1* gene, is mutated in neurofibromatosis 1, one of the most common genetic diseases. Oral manifestations are common and a high prevalence of hyposalivation was recently described in individuals with neurofibromatosis 1. Although neurofibromin is ubiquitously expressed, its expression levels vary depending on the tissue type and developmental stage of the organism. The role of neurofibromin in the development, morphology, and physiology of salivary glands is unknown and a detailed expression of neurofibromin in human normal salivary glands has never been investigated.

**Aim:**

To investigate the expression levels and distribution of neurofibromin in acinar and ductal cells of major and minor salivary glands of adult individuals without NF1.

**Material and method:**

Ten samples of morphologically normal major and minor salivary glands (three samples of each gland: parotid, submandibular and minor salivary; and one sample of sublingual gland) from individuals without neurofibromatosis 1 were selected to assess neurofibromin expression through immunohistochemistry. Immunoquantification was performed by a digital method.

**Results:**

Neurofibromin was expressed in the cytoplasm of both serous and mucous acinar cells, as well as in ducts from all the samples of salivary glands. Staining intensity varied from mild to strong depending on the type of salivary gland and region (acini or ducts). Ducts had higher neurofibromin expression than acinar cells (*p* = 0.003). There was no statistical association between the expression of neurofibromin and the type of the salivary gland, considering acini (*p* = 0.09) or ducts (*p* = 0.50) of the four salivary glands (parotid, submandibular, minor salivary, and sublingual gland). Similar results were obtained comparing the acini (*p* = 0.35) and ducts (*p* = 0.50) of minor and major salivary glands. Besides, there was no correlation between the expression of neurofibromin and age (*p* = 0.08), and sex (*p* = 0.79) of the individuals, considering simultaneously the neurofibromin levels of acini and duct (*n* = 34).

**Conclusion:**

Neurofibromin is expressed in the cytoplasm of serous and mucous acinar cells, and ductal cells of salivary glands, suggesting that this protein is important for salivary gland function.

## Introduction

Neurofibromatosis 1 (NF1, OMIM 162,200) is one of the most frequent genetic diseases in humans and has an autosomal dominant pattern [1–3]. NF1 is caused by mutations in the *NF1* gene, which encodes the neurofibromin protein that functions, in part, as a negative regulator of the Ras protein [2]. Therefore, neurofibromin is classified as a tumor suppressor protein.

Although neurofibromin is ubiquitously expressed, its expression levels vary according to the tissue type and developmental stage [4]. Neurofibromin is highly expressed in adult neurons, Schwann cells, astrocytes, oligodendrocytes, and leukocytes [5–7]. It is weakly expressed in other cells, such as osteoblasts and chondrocytes [8].

Neurofibromin is located in the cytoplasm, but can also be found in the nucleus and associated with plasma membrane microdomains [9, 10]. Therefore, neurofibromin is considered a multifunctional protein that affects several cellular processes in cells of different tissues [11].

Individuals with NF1 can present a variety of clinical manifestations, mainly multiple neurofibromas, café-au-lait spots, freckle-like lesions, Lisch nodules, and bone deformities [12]. Oral manifestations in soft and hard tissues occur in up to 92 % of NF1 individuals [13]. Increased fungiform papillae of the tongue and neurofibromas are the main manifestations in oral soft tissues [14–16]. The most common radiographic findings are the enlargement of the mandibular foramen, mental foramen, and mandibular canal [17]. Moreover, alterations in the coronoid process and mandibular condyle are commonly observed [13, 17]. Recently, in our previous study, it was showed that 59 % of the NF1 individuals have hyposalivation, presenting a higher prevalence compared with a control group (22 %), paired by sex and age [18]. None of the external factors related to hyposalivation (medication, smoking, low fluid intake, caffeinated and alcoholic beverages) was associated with a low salivary flow rate in individuals with NF1. Therefore, these previous results suggest that alterations in neurofibromin due to mutations in *NF1* gene, that occur in individuals with NF1, may be related to a reduction of salivary glands function. Nevertheless, the role of neurofibromin in the development, morphology, and physiology of salivary glands is unknown and a detailed expression of neurofibromin in human salivary glands has never been investigated.

The hypothesis of this study is that neurofibromin is highly expressed in human normal salivary gland tissues. The aim was to investigate the expression levels, distribution, and location of neurofibromin in acinar and ductal cells of adult major and minor salivary glands of individuals without NF1.

## Materials and methods

This work was approved by the research ethics committee of Hospital Universitário Antônio Pedro (HUAP) (protocol number #1,645,631), and the procedures performed following the Helsinki Declaration of 1975, as revised in 1983.

The study sample comprises morphologically normal tissues of major and minor salivary glands from patients without NF1 obtained from the pathology files of the Pathological Anatomy Service of HUAP - Universidade Federal Fluminense (UFF), Niterói, Rio de Janeiro, Brazil. All the cases of biopsy of major and minor salivary glands registered from 2010 to 2016 were evaluated. The inclusion criteria were: individuals over 18 years of age; presence of sufficient amount of morphologically normal salivary gland tissue for the construction of the Tissue Macroarray (TMaA) paraffin blocks; pathological request-form adequately filled. Cases of patients with NF1 were excluded based on the information obtained from the medical records.

Three samples of parotid, submandibular and minor salivary glands, and one sample of sublingual gland from individuals without NF1 were selected to assess neurofibromin expression through immunohistochemistry. Two minor salivary glands were biopsied to investigate Sjögren syndrome, however, there was no morphological alteration related to the syndrome in the samples, and the diagnosis of Sjögren syndrome was not either confirmed through the histopathological exam or by the investigation of other diagnosis criteria [19]. The other sample of minor salivary glands was removed together with a mucocele (mucous extravasation phenomena), and the major salivary glands were excised due to the presence of benign tumors. Clinical data are shown in Table 1.

**Table 1:** Clinical data

Gland type	Age	Sex
Minor	12 years-old	male
Minor	22 years-old	male
Minor	42 years-old	male
Sublingual	39 years-old	male
Submandibular	32 years-old	female
Submandibular	50 years-old	female
Submandibular	64 years-old	female
Parotid	41 years-old	male
Parotid	62 years-old	male
Parotid	36 years-old	female

For the analysis of the expression of neurofibromin, four Tissue Macroarray (TMaA) paraffin blocks were constructed as previously described [20]. From each donor paraffin block of parotid, submandibular and sublingual glands, three or four morphologically normal areas containing serous and mucous acini, and intercalated, striated, and excretory ducts were selected. In cases of minor salivary glands, two or three entire morphologically normal glands were selected. The samples were removed from the donor paraffin blocks using a punch with 6 mm diameter.

Sections with 5 µm from the TMaA paraffin blocks were cut and collected on silane-coated slides. After dewaxing, neurofibromin was demonstrated immunohistochemically using HiDef Detection™ Polymer System (Cell Marque 6600, Rocklin CA - USA). Endogenous peroxidase activity was blocked by incubation for 30 minutes in 3 % H2O2 in distilled water at room temperature. Antigen retrieval was performed using an EDTA (ethylenediaminetetraacetic acid) solution, pH 9 (Thermo Scientific, Fremont CA - USA, ref AP-9003-500) for 20 minutes in a water bath. Afterward, the glass slides were kept at room temperature in the same solution for 20 minutes. Non-specific protein binding was blocked with skimmed milk powder diluted in Tris solution with NaCl and BSA (bovine serum albumin) for 15 minutes in a humid chamber at room temperature. Sections were incubated with a 1:50 dilution of the primary antibody (anti-neurofibromin a mouse monoclonal antibody anti-neurofibromin; clone McNFn27b, code sc-20,016; Santa Cruz Biotechnology, USA) for 16 hours at 4 °C. Visualization was performed by incubation for 5 min in diaminobenzidine. The sections were then counterstained with Harris’ hematoxylin and coverslipped with Entellan (code 107,961; Merck, Frankfurt, Germany). Between each step, sections were washed three times for 10 min in Tris-buffered saline. All incubations were carried out in humidified chambers to prevent evaporation. The negative control was performed by omission of the primary antibody and a sample of a peripheral nerve was used as a positive control of the reaction.

**Fig. 1:**
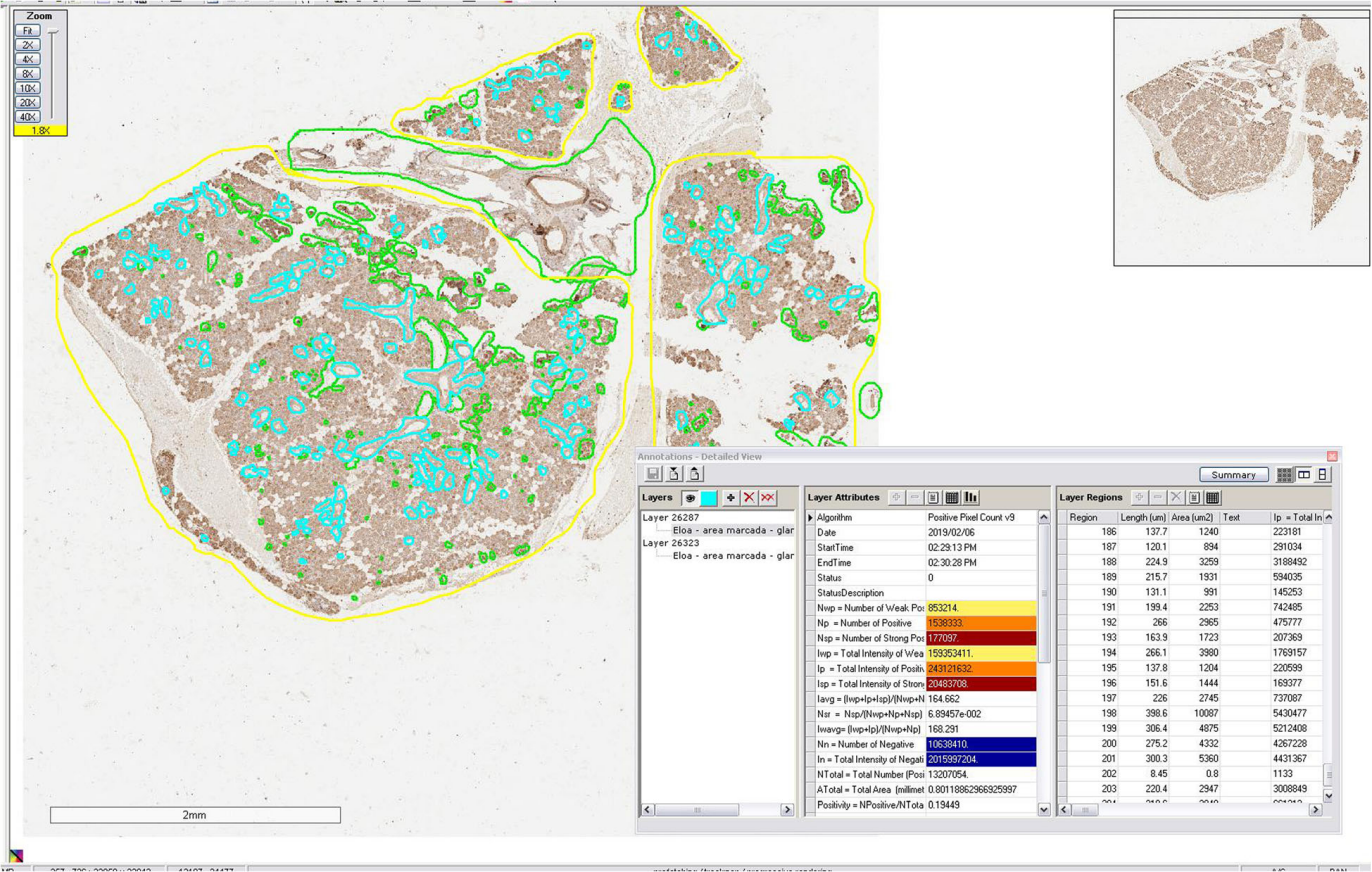
Marked areas of glandular tissue using ImageScope™ software. Yellow: glandular tissue; blue: salivary ducts; green: excluded areas. Scale bar: 2 mm. Microscope objective used for scanning: ×40

**Fig. 2:**
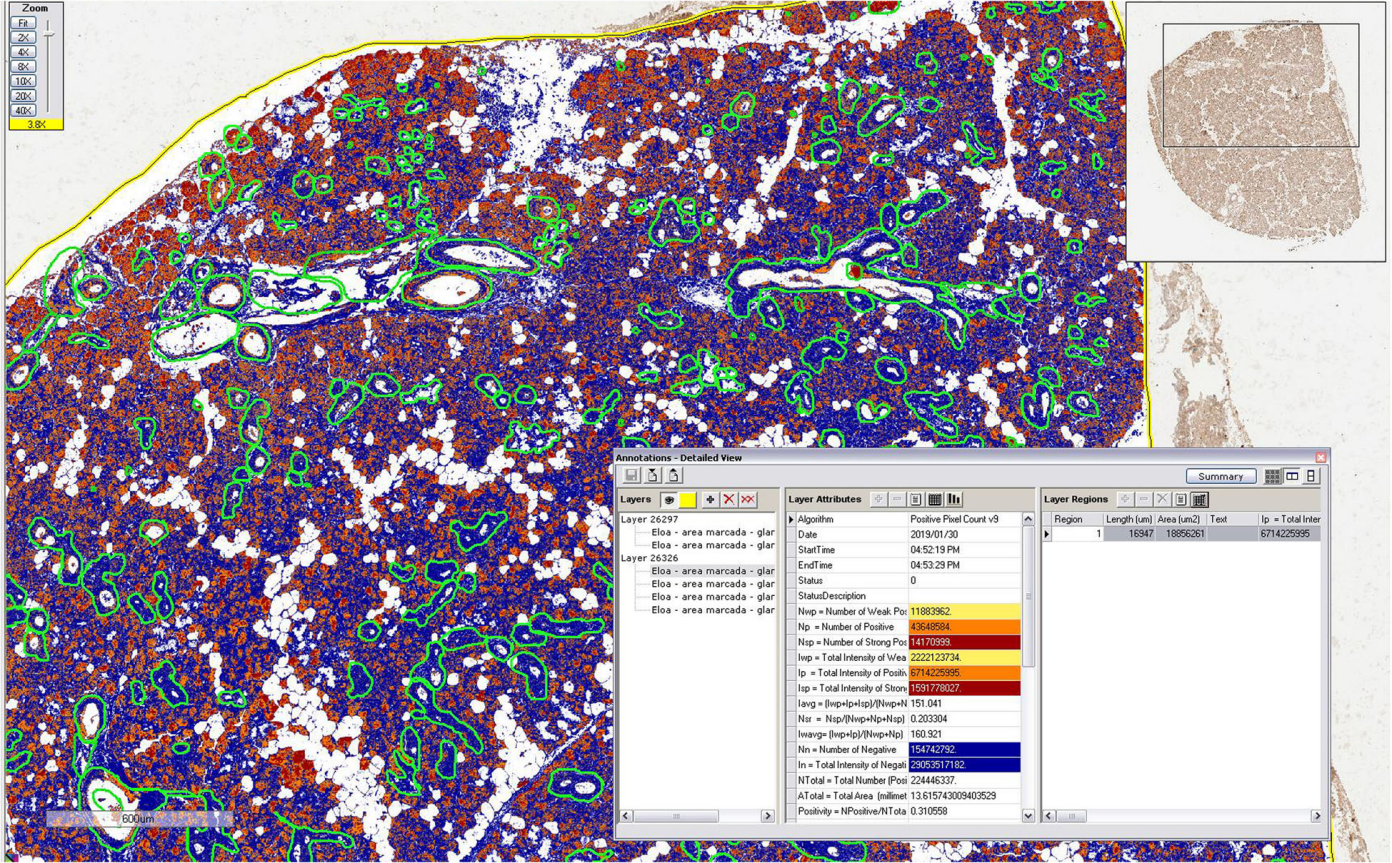
A salivary gland sample with immunoquantification generated after analysis in Spectrum™ software. Scale bar: 600 μm. Microscope objective used for scanning: ×40

**Fig. 3:**
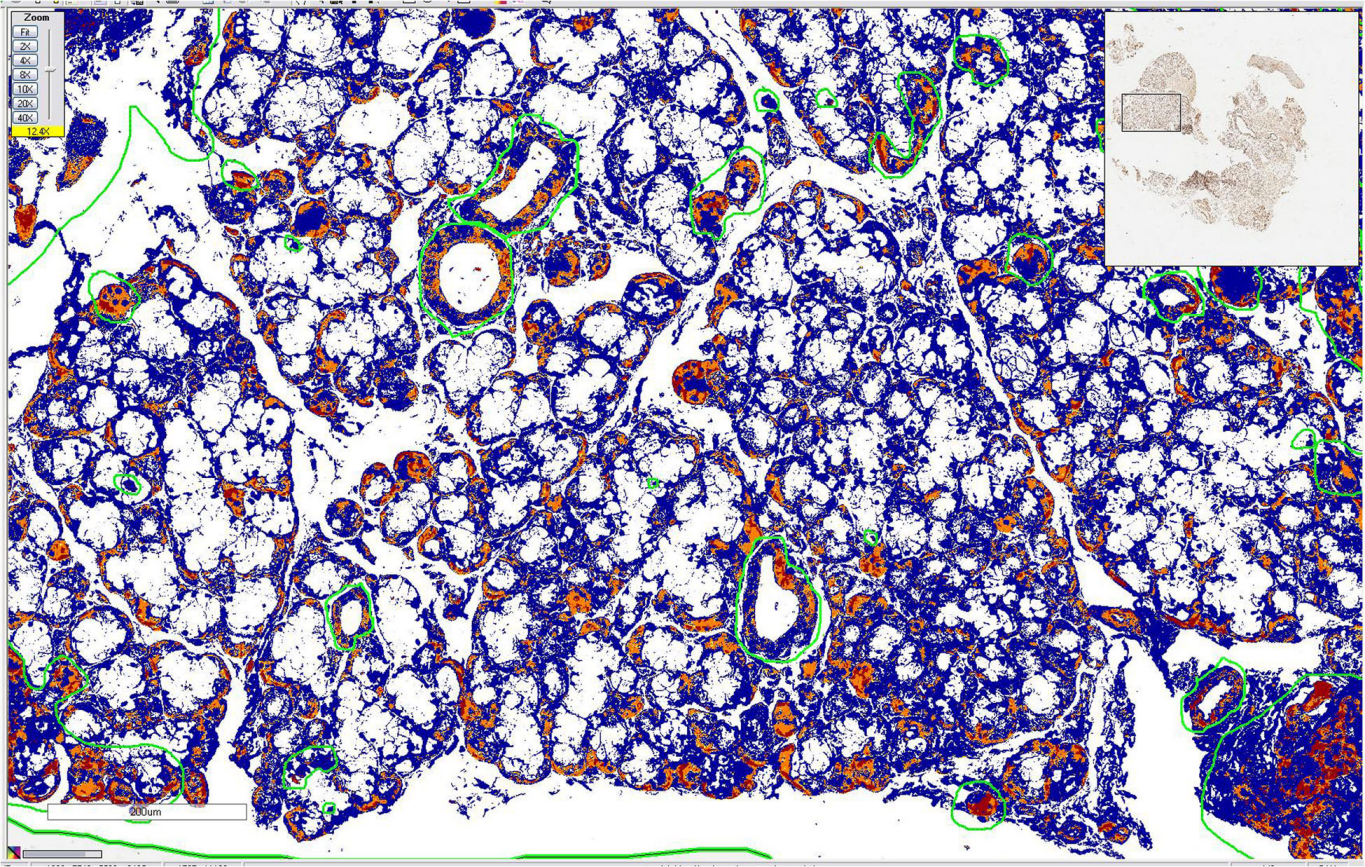
Marked area after analysis with the pixel count macro of a salivary gland sample. Blue: area with negativity; yellow: area with weak positivity; orange: area with moderate positivity; red: area with strong positivity. Scale bar: 200 µm. Microscope objective used for scanning: ×40

Aperio Digital Pathology® System (Leica Biosystems, Wetzlar, Germany) was used for immunoquantification. TMaA slides were scanned (using a x40 microscope objective) and organized in TMAlab™ software. The areas of acini and ducts were selected for analysis. Regions with large blood vessels and artifacts (e.g. tissue folding) were excluded using the ImageScope™ software (Fig. 1). After calibration (in ScanScope™ software), immunoquantification was performed with Spectrum™ digital pathology information management software through evaluation of the brown (positive) and blue (negative) staining, using the Positive Pixel Count algorithm (Fig. 2). Neurofibromin expression was showed as positive area percentage. For the analysis of staining intensity, the algorithm was able to classify each pixel as 0 (negative, threshold 256–220), 1 (weak positive staining, threshold 221–175), 2 (moderate positive staining, threshold 176–100), or 3 (strong positive staining, threshold 99–0) (Fig. 3).

## Results

**Fig. 4:**
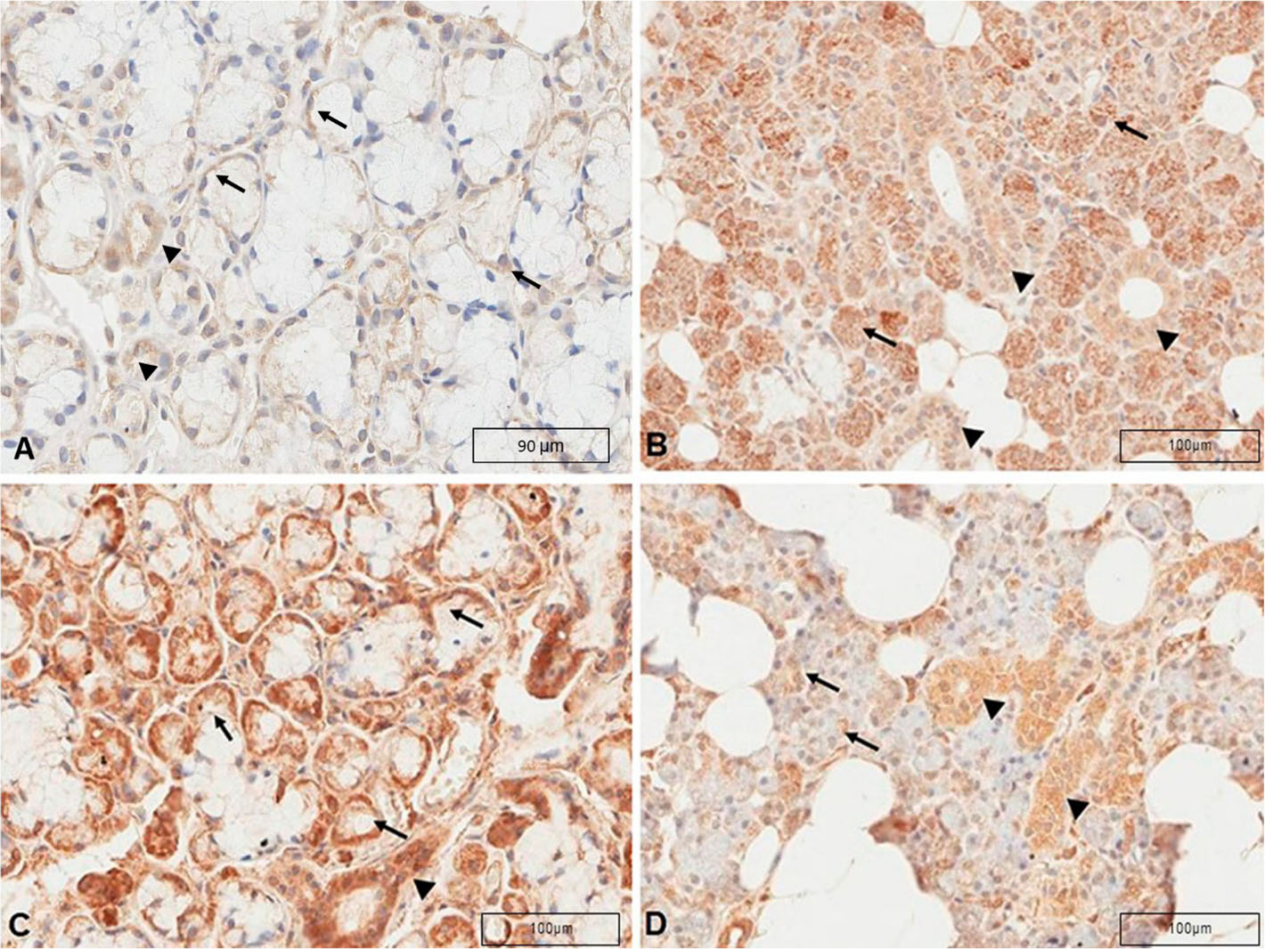
Neurofibromin immunostaining in salivary glands. **a** Minor salivary gland. Weak to moderate cytoplasm positivity for neurofibromin in the apical portion of the mucous acinar cells (arrows) and cytoplasm moderate positivity in the ductal cells (arrowheads). Scale bar: 90 µm. **b** Submandibular gland. Moderate to strong positivity for neurofibromin in the cytoplasm of the serous acinar cells and apical portions of the mucous acinar cells (arrows). Moderate positivity in the cytoplasm of the ductal cells (arrowheads). Scale bar: 100 µm. **c** Sublingual gland. Moderate to strong positivity for neurofibromin in the cytoplasm of the serous acinar cells (arrows) and moderate positivity in the cytoplasm of the ductal cells (arrowheads). Scale bar: 100 µm. **d** Parotid gland. Weak positivity for neurofibromin in the cytoplasm of the apical portion of the serous acinar cells (arrows). Moderate positivity in the cytoplasm of the ductal cells. (arrowheads). Scale bar: 2mm. Microscope objective used for scanning: ×40

Neurofibromin was expressed in the cytoplasm of serous and mucous acinar cells, as well as in the ductal cells from all the samples of glands (Fig. 4; Table 2). Neurofibromin had a higher expression level in ducts than acini (Wilcoxon signed-sum test; *p* = 0.003). There was no statistical association between the expression of neurofibromin and the type of the salivary gland, considering the acini (Mann-Whitney test; *p* = 0.09; *n* = 17) or ducts (Mann-Whitney test; *p* = 0.50; *n* = 17) of the four types of salivary glands (parotid, submandibular, minor salivary and sublingual gland). Similar results were obtained comparing the acini (Mann-Whitney test; *p* = 0.35; *n* = 17) and ducts (Mann-Whitney test: *p* = 0.50; *n* = 17) of minor and major salivary glands. There was no correlation between the expression of neurofibromin and age (Spearman’s rho correlation; *p* = 0.08, coefficient rho = 0.3), and sex (Mann-Whitney test; *p* = 0.79) of the individuals, considering simultaneously the neurofibromin levels of acini and duct (*n* = 34).

**Table 2:** Area data and immunostaining positivity after analysis with the pixel count macro

	n	Average area (mm^2^)	Positivity (average)	Standard deviation (+)
Ducts				
Minor	3	17.65	57%	0.36
Sublingual	1	8.92	35%	−
Submandibular	3	2497.02	52%	0.05
Parotid	3	7398.31	66%	0.06
Acini				
Minor	3	4295.20	49%	0.29
Sublingual	1	18856.26	31%	-
Submandibular	3	6513.87	47%	0.05
Parotid	3	15303.89	23%	0.06

### Minor salivary glands

In minor salivary glands, weak to moderate positivity for neurofibromin was observed in the apical portion of the mucous acinar cells (Fig. 4a). Moderate positivity was seen in the cytoplasm of ductal cells. Neurofibromin was expressed in 57 % of the total area of the ducts and 49 % of the total area of acini.

### Submandibular glands

In the submandibular glands, moderate to strong positivity for neurofibromin was found throughout the cytoplasm of the serous acinar cells (Fig. 4b). Moderate positivity was seen in the cytoplasm of the ductal cells. Neurofibromin was expressed in an average of 47 % of the total area of the acini and 52 % of the total area of the ducts.

### Sublingual glands

In the sublingual gland, moderate to strong positivity for neurofibromin was found throughout the cytoplasm of the serous acinar cells and in the apical portion of the mucous acinar cells (Fig. 4c). Moderate positivity was seen in the cytoplasm of the ductal cells. Neurofibromin was expressed in 31 % of the total area of the acini and 35 % of the total area of the ducts.

### Parotid glands

In the parotid glands, weak positivity for neurofibromin was found in the cytoplasm of the apical portion of the serous acinar cells (Fig. 4d). Moderate positivity was seen in the cytoplasm of the ductal cells. Neurofibromin was expressed in 23 % of the total area of the acini and 66 % of the total area of the ducts.

## Discussion

Kimura et al. [21], in an immunohistochemical study on pheochromocytoma and tumors associated with NF1, found expression of neurofibromin in the ducts of a salivary gland used as a control. Nevertheless, there are no details on the expression of neurofibromin neither which type of salivary gland was investigated. To the best of our knowledge there are no other studies on the expression of neurofibromin in normal salivary glands tissues.

This study is the first to show, using an antibody that detects all the five known isoforms of neurofibromin, that this protein is expressed in the cytoplasm of all types of adult salivary glands, in mucous and serous acinar cells, as well as in the ductal cells, and is independent of age and sex. Neurofibromin had a higher expression level in ducts than acini and there were no differences in the expression considering the different salivary glands. Depending on the type of salivary gland and location (ducts or acini), neurofibromin expression varied from weak to strong.

Neurofibromin is a large protein that presents a GTPase-activating protein (GAP) related domain (GRD) [11]. Therefore, as other GAP proteins, one of its functions is to accelerate the hydrolysis of Ras-GTP to Ras-GDP, converting the active GTP (guanosine triphosphate) bound form to an inactive GDP (guanosine diphosphate) bound form, and thereby negatively regulating the Ras signal [22]. Ras is a family of related proteins belonging to a class called small GTPases, involved in transmitting signals from growth factor receptors to various downstream signaling molecules that eventually alter gene expression in the nucleus, regulating many major cellular processes, such as cell proliferation and differentiation, migration, apoptosis, and morphology [23]. Neurofibromin has also a role in the development and homeostasis in many tissues [22]. Although the function of neurofibromin in the salivary glands’ development, morphology and function is largely unknown, its expression in salivary glands tissues found in this study, the high prevalence of hyposalivation in NF1 individuals, and its known interaction with other proteins involved in embryogenesis and physiology of the salivary glands, suggests that neurofibromin present an important role in these processes.

There is a complex signaling cross-talks between neurofibromin and other members of the superfamily of small GTPases, including Rho binding domain [24]. Rho GTPases play a crucial role in the morphogenesis of the salivary gland lumen in animal models and acinus formation in human salivary glands, [25, 26] and the small GTPase Rac is involved in the regulation of salivary gland migration during its morphogenesis [27]. Moreover, neurofibromin is involved in cytoskeletal organization and cell motility through regulation of the Rho-ROCK-LIMK2-cofilin pathway [24, 28]. Coordinated regulation of actin cytoskeleton dynamics is required for several fundamental cellular events such as cell movement, cell junction, and cytokinesis [29]. Salivary proteins are secreted by exocytosis (i.e., the fusion between secretory granule membranes and the apical plasma membrane of salivary acinar cells) [30]. Studies indicate that the actin cytoskeleton, which is localized under the plasma membrane, prevents secretory granules from reaching their exocytotic destination [31, 32]. Therefore, breakdown and reorganization of the actin cytoskeleton are necessary for exocytosis. Also, the polarization and the cellular junctions allow the movement of the salivary components to the lumen of the structure and guarantee the impermeability of the duct cells [33]. Moreover, Rho GTPases have emerged as key mediators of Wnt signals, which are important for salivary gland cells migration during embryogenesis [34]. In adult salivary glands, expression of the Wnt signaling is maintained in the ductal epithelium for promoting regeneration post-injury or expansion of progenitor cells by inhibiting apoptosis and preserving normal tissue functions [35].

More studies are necessary to understand the role of neurofibromin in the development and function of salivary glands. Future studies should investigate the impact of the alterations in neurofibromin due to mutations in the *NF1* gene, that occurs in individuals with NF1, in the development, morphology and physiology of salivary glands to better understand the relationship between NF1 and hyposalivation.

## Conclusions

Neurofibromin is expressed in the cytoplasm of serous and mucous acinar cells, and ductal cells of adult salivary glands. The expression is higher in ductal cells of all types of salivary glands than in acini. The results suggest that this protein is important for the normal salivary gland function.

## Data Availability

The datasets used and/or analyzed during the current study are available from the corresponding author on reasonable request.

## Abbreviations

NF1: Neurofibromatosis 1
HUAP: Hospital Universitário Antônio Pedro
UFF: Universidade Federal Fluminense
HE: Hematoxylin-eosin
TMaA: Tissue Macroarray
EDTA: Ethylenediaminetetraacetic acid
BSA: Bovine serum albumin
GAP: GTPase-activating protein
GRD: GAP related domain
GTP: Guanosine triphosphate
GDP: Guanosine diphosphate
LIMK2: LIM kinase 2
